# Surgical management of a retro-rectal cystic hamartoma (tailgut cyst) using a trans-rectal approach: a case report and review of the literature

**DOI:** 10.1186/1752-1947-8-11

**Published:** 2014-01-06

**Authors:** Edvinas Kildušis, Narimantas Evaldas Samalavičius

**Affiliations:** 1 Center of Oncosurgery, Institute of Oncology, Vilnius University, Santariskiu Street 1, Vilnius LT-08660, Lithuania; 2 Clinic of Internal Medicine, Family Medicine and Oncology, Medical Faculty of Vilnius University, MK Ciurlionio Street 21, Vilnius LT-03101, Lithuania

**Keywords:** Retro-rectal space, Retro-rectal cystic hamartoma, Tailgut cyst, Trans-rectal approach, Surgical management

## Abstract

**Introduction:**

Retro-rectal cystic hamartoma (tailgut cyst) is a rare congenital developmental lesion arising from post-natal primitive gut remnants in the retro-rectal space. The rarity of the lesion and its anatomical position usually leads to difficulty in diagnosis and surgical management. Complete surgical resection remains the cornerstone of treatment. A dozen or so surgical approaches have been described in the literature to date to make the operation as simple as possible, but the trans-rectal access route is extremely rarely reported and not well described. Here, we present a case that demonstrates the trans-rectal approach to a retro-rectal tumor is a feasible option in terms of surgical radicality, minimal invasiveness and safety for carefully selected patients with this rare type of retro-rectal cystic lesion.

**Case presentation:**

A 29-year-old Caucasian woman was referred to our institution due to perineal pain extending to the right inguinal region. Her symptoms had been present for the last two months. She was first examined at her regional hospital for a suspected ruptured ovarian cyst; however, after consultation with a gynecologist and a computed tomography scan of her pelvis, a tumor in the retro-rectal space was discovered. Our patient was admitted to our hospital and when a pelvic magnetic resonance imaging study confirmed the diagnosis of the retro-rectal cystic formation, a complete extirpation of retro-rectal tumor fixed to the coccyx using trans-rectal approach was performed. The final pathological diagnosis was retro-rectal cystic hamartoma (tailgut cyst) with no evidence of malignancy. Her post-operative course was uneventful, and at four months after surgery our patient is symptom free with no evidence of recurrent or residual disease.

**Conclusions:**

Trans-rectal excision is feasible in terms of surgical radicality and is a simple to perform, minimally invasive and safe option, providing complete recovery for carefully selected patients with retro-rectal cystic hamartoma treated operatively.

## Introduction

Retro-rectal cystic hamartoma, also known as tailgut cyst, is an uncommon congenital developmental lesion arising from post-natal primitive gut remnants, generally located in the retro-rectal space [1,2]. The anatomical position and rarity of the lesion leads to difficulty firstly in diagnosis (the lesion is often misdiagnosed) and secondly in surgical management (the condition is often suboptimally managed) [3]. Furthermore, retro-rectal lesions in women can mimic gynecological pathology, and the risk of malignant transformation of a tailgut cyst always exists. Despite that, the role of pre-operative biopsy for retro-rectal tumors is very controversial [4], but most authors agree that it can be a more harmful than useful option. This is why pre-operative high-resolution modern imaging techniques (pelvic computed tomography (CT) or magnetic resonance imaging (MRI)) play such a crucial role in differential diagnostics between retro-rectal tumors and planning the surgical management of retro-rectal lesions, including tailgut cysts. Complete surgical resection with negative margins still remains the cornerstone of surgical treatment, as this eliminates the potential of recurrence, hemorrhage, infection, compression and malignant changes [2]. A huge variety of surgical approaches have been described in the literature to ease the operation. The abdominal or anterior, the trans-sacral or posterior and the combined abdomino-sacral approaches are well described in the literature, but others such as the trans-vaginal or trans-anorectal access paths have only been reported extremely rarely [5]. The literature on the trans-rectal approach is limited to case reports. Therefore, in view of the scarce information available on this matter, we present a case demonstrating the trans-rectal approach to retro-rectal tumor as a feasible option in terms of surgical radicality, minimal invasiveness and safety for carefully selected patients with this rare type of retro-rectal cystic lesion.

## Case presentation

**Figure 1:**
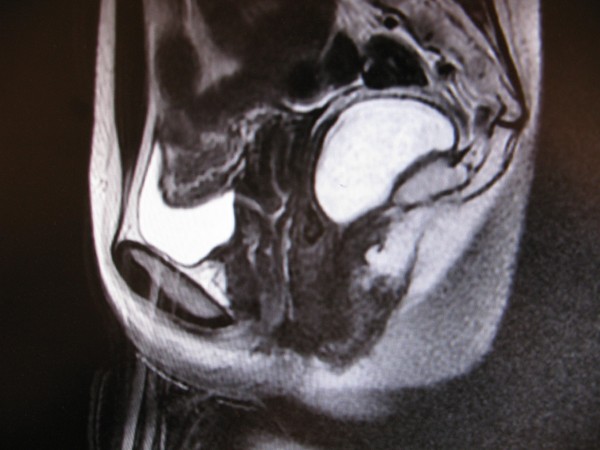
High-resolution modern imaging view of retro-rectal cystic hamartoma.

**Figure 2:**
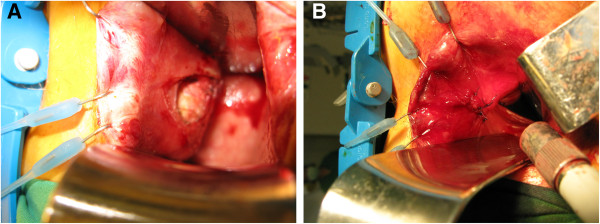
Mobilization of the retro-rectal cystic hamartoma (A) and reconstruction of the rectal wall (B).

**Figure 3:**
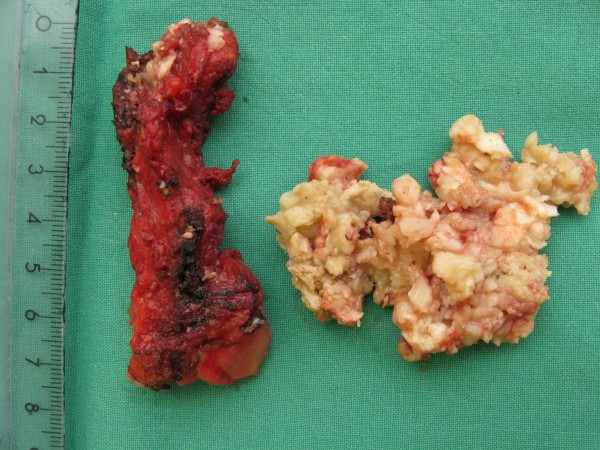
Retro-rectal cystic hamartoma.

A 29-year-old Caucasian woman was referred to our institution due to perineal pain extending to the right inguinal region. Her symptoms had been present for the last two months. Previously, she had been examined in a regional hospital, where a ruptured ovarian cyst was suspected. After gynecologist consultation and a pelvic CT scan, a tumor in her retro-rectal space was discovered. Our patient was admitted to our hospital for further examination and treatment. On digital rectal examination a lesion at the projection from four o’clock to eight o’clock in the lower rectum behind the rectal wall was palpated, with the lower boarder extending to as low as 2cm above the dentate line. A pelvic MRI scan detected a large cystic formation (7×5×4cm) in the retro-rectal space (slightly more to the right) (Figure 1). The complete excision of retro-rectal tumor (which was fixed to the apex of the coccyx) using a trans-rectal approach was performed (Figure 2A,B) and the final pathological diagnosis was retro-rectal cystic hamartoma (tailgut cyst) with no evidence of malignancy (Figure 3). Her post-operative course was uneventful and at four months after surgery our patient is symptom free with no evidence of recurrent or residual disease.

## Discussion

The retro-rectal (pre-sacral) space is the virtual anatomic region bounded by the rectum (mesorectum, fascia propria of the rectum) anteriorly, the sacrum (pre-sacral fascia) posteriorly, the peritoneal reflection (forms the roof of the pre-sacral space) superiorly and the levator ani and coccygeus muscles (fusion of the pre-sacral parietal fascia and the rectal visceral fascia) inferiorly [6-10]. The lateral boundaries are the endopelvic fascia (lateral ligaments), the ureters and the iliac vessels [5,10]. Anatomically, the pre-sacral space is divided into superior and inferior portions by recto-sacral fascia (Waldeyer) and contains loose connective tissue [10].

Tumors of the retro-rectal (pre-sacral) space in the adult population are uncommon and even rare [11]. These tumors are classified according to predominant cell line type and are divided into congenital (55 percent to 65 percent), neurogenic (10 percent to 12 percent), inflammatory (5 percent), osseous (5 percent to 11 percent) and miscellaneous (12 percent to 16 percent) [11,12]. It is known that developmental cysts account for approximately 60 percent of congenital retro-rectal tumors [7] and must be differentiated from cystic sacrococcygeal teratoma, anterior sacral meningocele, anal duct or gland cyst, necrotic rectal leiomyosarcoma, and necrotic sacral chordoma [13]. They are more common in middle-aged (40 to 60 years old) women (the female-to-male ratio is 3:1) [6,13] and arise from aberrant remnants of the post-anal primitive gut in cases of incomplete embryogenetic involution [7]. Depending on the embryonic cell of origin, these cysts can be classified into epidermoid cysts, dermoid cysts, neurenteric cysts, teratomas and enteric cysts (retro-rectal cysts and cystic rectal duplication) [7,13]. Retro-rectal cysts have been described in the literature under different names such as tailgut cyst, cyst of post-anal intestine, mucus-secreting cyst, enterogenous cyst, simple cyst, myoepithelial hamartoma of the rectum and retro-rectal cystic hamartoma. Tailgut cysts arise from hindgut embryonic remnants and can be lined by various epithelia, but typically by glandular or transitional epithelium [6,13].

There is a lack of information on retro-rectal cystic hamartoma natural history and biological behavior [7]; only limited reports exist. Tailgut cysts are usually asymptomatic in adults. Symptoms only occur due to the local mass effect on surrounding organs (rectal fullness, constipation, painful defecation, lower abdominal and/or back pain or genitourinary obstruction (dysuria)), infection (cysts with secondary infection have typical symptoms of anorectal or pelvic abscess and fistula, or peri-anal sinus [7]), bleeding or malignant transformation/degeneration (pain in the anorectal region) [11,13]. Retro-rectal cystic hamartomas in the pre-sacral space are usually well defined, thin-walled and multicystic or unilocular [13]. Despite the fact that the majority of tailgut cysts are benign, the current literature shows an increasing risk of malignant transformation [7,13] when the most common histopathologic diagnoses are adenocarcinoma or carcinoid [13]. Interestingly, malignancy is more common in men due to delay in diagnosis; this is because among women the cystic lesions are frequently detected during gynecological examinations [9].

Half of developmental cysts are diagnosed incidentally during examination for other conditions [7]. Diagnosis requires high-resolution modern imaging techniques (pelvic MRI or CT) [7] as the standard for pre-operative evaluation of retro-rectal cysts [3], which helps to avoid the need for pre-operative biopsy in the following ways. A CT scan of the pelvis helps to distinguish cystic, solid or mixed lesions and assess for sacral involvement or invasion to adjacent structures. Magnetic resonance imaging has higher resolution in soft tissue and visualizes soft-tissue planes, helps to evaluate the relationships of the lesions with bones, muscles and nerves. MRI findings usually correlate slightly more with the final histology of the tumor than CT scan results. Of course, primarily patients must undergo a digital rectal examination and colonoscopy (and additionally, for women, a gynecological examination) to exclude more common conditions [3,9]. Final diagnosis, of course, remains histopathologic [13], but most authors agree that a pre-operative biopsy of the tumor is not indicated because it can be uninformative and the risk of bleeding, infection and dissemination of malignant cells into the peritoneal cavity or seeding of the biopsy tract is high [7,11]. Complete surgical excision of the epithelial lining with clear resection margins remains the cornerstone option for surgical management of tumors in the retro-rectal space [7,11,13], even if asymptomatic, but treatment may be very challenging when cystic lesions are associated with a fistula or abscess and especially when adjacent structures are involved (sacrum, rectum, blood vessels and nerves) or when the disease is not correctly diagnosed at presentation [7,9]. Furthermore, the risks of recurrence, hemorrhage, chronic infection and malignant degeneration to adenocarcinoma or squamous carcinoma always exists [13].

It is postulated that the best treatment for retro-rectal tumors remains complete surgical excision of the retro-rectal mass (cyst) as mentioned above, which eliminates the problems of potential recurrence, hemorrhage, infection, compression and the possibility of malignant change [2,6]. Historically, the classical treatment in this area consists of three different approaches: the anterior (trans-abdominal), the posterior approaches (inter-sphincteric, trans-sphincteric parasacrococcygeal, trans-sacral, trans-sacrococcygeal, trans-anorectal and trans-vaginal) or a combination of both [7,11]. Of course, the selection of approach is key to successful treatment and is determined by the nature (morphology), localization, size of the retro-rectal lesion and its relationship with adjacent structures. It is argued that the posterior approach is required when the retro-rectal mass is below S3 or the sacral promontory, and the anterior (abdominal) approach should be reserved for relatively high lesions (above S3 or the sacral promontory) [11]. Most authors report that trans-anorectal excisions are usually preferred in cases of relatively small, non-infected and low-lying lesions. The other or multiple approaches are used in complicated cases, such as infected cysts (with concomitant perineal fistulas), when inflammation persist or adjacent structures and organs are involved, or when symptoms of malignant transformation (degeneration) appear [3,7]. By the anterior approach, the surgeon achieves good direct visualization of pelvic structures, iliac vessels and ureters. The posterior approach gives good access to the caudal component [3], but the major disadvantages are the absence of control over pelvic vessels and the potential for injury to the lateral pelvic nerves [5]. The mini-invasive laparoscopic trans-abdominal approach has been described in the literature too, as well as the trans-anal endoscopic microsurgery technique [11]. The laparoscopic approach offers benefits such as an excellent visualization of the pre-sacral space and its content and reduced surgical trauma [7]. The trans-anal endoscopic microsurgery method is usually associated with low incidence of morbidity [11]. However, both of these modern techniques require additional equipment, and of course cost. Unfortunately, there are no long-term studies that compare these latter two methods with our approach. Therefore, because of the extreme rarity of the tailgut cyst and the consequential scarce information on surgical treatment of this type of retro-rectal lesion, especially through the trans-rectal approach (other approaches in the literature are described in more detail), we report a representative case of successful surgical management of retro-rectal cystic hamartoma using the above-mentioned approach. Furthermore, we advocate the trans-rectal technique as a feasible, easy to perform, minimally invasive and safe option for treating relatively low-lying, non-complicated and benign retro-rectal lesions that requires no additional cost and is associated with very low predicted morbidity.

## Conclusions

The prognosis of surgical management depends on achieving complete surgical excision of the tailgut cyst with clear margins. Trans-rectal excision is feasible in term of surgical radicality and can be an easy to perform, minimally invasive and safe option, providing complete recovery for carefully selected patients with retro-rectal cystic hamartoma treated operationally.

## Consent

Written informed consent was obtained from the patient for publication of this case report and any accompanying images. A copy of the written consent is available for review by the Editor-in-Chief of this journal.

## Competing interests

The authors declare that they have no competing interests.

## Authors’ contributions

EK and NES examined and treated our patient, and analyzed and interpreted the data from our patient. Both authors read and approved the manuscript.

## References

[B1] Smit R Gregorini D Beltrán R Martorelli J Granada G Lespi P Retrorectal cyst hamartoma: report of a pediatric case Arch Argent Pediatr 2010 108 82 85 20544128 10.1590/S0325-00752010000300015

[B2] Lim SW Huh JW Kim YJ Kim HR Laparoscopy-assisted resection of tailgut cysts: report of a case Case Rep Gastroenterol 2011 14 22 27 10.1159/000322912 PMC3037990 21326854

[B3] Lin C Jin K Lan H Teng L Lin J Chen W Surgical management of retrorectal tumors: a retrospective study of a 9-year experience in a single institution Onco Targets Ther 2011 4 203 208 22162926 10.2147/OTT.S25271 PMC3233279

[B4] Neale JA Retrorectal tumors Clin Colon Rectal Surg 2011 24 149 160 10.1055/s-0031-1285999 22942797 PMC3311502

[B5] Aranda-Narváez JM González-Sánchez AJ Montiel-Casado C Sánchez-Pérez B Jiménez-Mazure C Valle-Carbajo M Santoyo-Santoyo J Posterior approach (Kraske procedure) for surgical treatment of presacral tumors World J Gastrointest Surg 2012 27 126 130 10.4240/wjgs.v4.i5.126 PMC3364338 22655127

[B6] Sriganeshan V Alexis JB A 37-year-old woman with a presacral mass. Tailgut cyst (retrorectal cystic hamartoma) Arch Pathol Lab Med 2006 130 77 78 10.5858/2006-130-e77-AYWWAP 16683902

[B7] Rosa G Lolli P Vergine M El-Dalati G Malleo G Surgical excision of developmental retrorectal cysts: results with long-term follow-up from a single institution Updates Surg 2012 64 279 284 10.1007/s13304-012-0168-x 22864760

[B8] Macafee DA Sagar PM El-Khoury T Hyland R Retrorectal tumours: optimization of surgical approach and outcome Colorectal Dis 2012 14 1411 1417 10.1111/j.1463-1318.2012.02994.x 22339762

[B9] Boscà A Pous S Artés MJ Gómez F Granero Castro P García-Granero E Tumours of the retrorectal space: management and outcome of a heterogeneous group of diseases Colorectal Dis 2012 14 1418 1423 10.1111/j.1463-1318.2012.03016.x 22390258

[B10] García-Armengol J García-Botello S Martinez-Soriano F Roig JV Lledó S Review of the anatomic concepts in relation to the retrorectal space and endopelvic fascia: Waldeyer's fascia and the rectosacral fascia Colorectal Dis 2008 10 298 302 10.1111/j.1463-1318.2007.01472.x 18257849

[B11] Serra Aracil X Gómez Díaz C Bombardó Junca J Mora López L Alcántara Moral M Ayguavives Garnica I Navarro Soto S Surgical excision of retrorectal tumour using transanal endoscopic microsurgery Colorectal Dis 2010 12 594 595 19906055 10.1111/j.1463-1318.2009.02126.x

[B12] Glasgow SC Birnbaum EH Lowney JK Fleshman JW Kodner IJ Mutch DG Lewin S Mutch MG Dietz DW Retrorectal tumors: a diagnostic and therapeutic challenge Dis Colon Rectum 2005 48 1581 1587 10.1007/s10350-005-0048-2 15937630

[B13] Doyle D Wyse G Casey M Kelly D Answer to case of the month #109. Retrorectal cystic hamartoma Can Assoc Radiol J 2006 57 179 181 16881476

